# POPcorn: An Online Resource Providing Access to Distributed and Diverse Maize Project Data

**DOI:** 10.1155/2011/923035

**Published:** 2011-12-27

**Authors:** Ethalinda K. S. Cannon, Scott M. Birkett, Bremen L. Braun, Sateesh Kodavali, Douglas M. Jennewein, Alper Yilmaz, Valentin Antonescu, Corina Antonescu, Lisa C. Harper, Jack M. Gardiner, Mary L. Schaeffer, Darwin A. Campbell, Carson M. Andorf, Destri Andorf, Damon Lisch, Karen E. Koch, Donald R. McCarty, John Quackenbush, Erich Grotewold, Carol M. Lushbough, Taner Z. Sen, Carolyn J. Lawrence

**Affiliations:** ^1^Department of Genetics, Development and Cell Biology, Iowa State University, Ames, IA 50011, USA; ^2^USDA-ARS Corn Insects and Crop Genetics Research Unit, Iowa State University, Ames, IA 50011, USA; ^3^Department of Computer Science, University of South Dakota, Vermillion, SD 57069, USA; ^4^Plant Biotechnology Center and Department of Molecular Genetics, The Ohio State University, Columbus, OH 43210, USA; ^5^Department of Biostatistics and Computational Biology and Department of Cancer Biology, Dana-Farber Cancer Institute, 450 Brookline Avenue, Sm822, Boston, MA 02215, USA; ^6^USDA-ARS Plant Gene Expression Center, Albany, CA 94710, USA; ^7^Department of Molecular and Cell Biology, University of California, Berkeley, CA 94720, USA; ^8^School of Plant Sciences, University of Arizona, Tucson, AZ 85721, USA; ^9^USDA-ARS Plant Genetics Research Unit, University of Missouri, Columbia, MO 65211, USA; ^10^Division of Plant Sciences, Department of Agronomy, University of Missouri, Columbia, MO 65211, USA; ^11^Department of Plant and Microbial Biology, University of California, Berkeley, CA 94720, USA; ^12^Horticultural Sciences Department, University of Florida, Gainesville, FL 32611, USA

## Abstract

The purpose of the online resource presented here, POPcorn (Project Portal for corn), is to enhance accessibility of maize genetic and genomic resources for plant biologists. Currently, many online locations are difficult to find, some are best searched independently, and individual project websites often degrade over time—sometimes disappearing entirely. The POPcorn site makes available (1) a centralized, web-accessible resource to search and browse descriptions of ongoing maize genomics projects, (2) a single, stand-alone tool that uses web Services and minimal data warehousing to search for sequence matches in online resources of diverse offsite projects, and (3) a set of tools that enables researchers to migrate their data to the long-term model organism database for maize genetic and genomic information: MaizeGDB. Examples demonstrating POPcorn's utility are provided herein.

## 1. Introduction

### 1.1. Need for the POPcorn Resource

In 1998, the National Science Foundation (NSF) launched the Plant Genome Research Program (PGRP), as part of the National Plant Genome Initiative. The establishment of PGRP coincided with an explosion of technologies that allowed large-scale genomic experiments to flourish, and PGRP grants fueled unprecedented advances in plant genomics research. This program was unique in that it strongly encouraged large collaborative projects and required project outcomes to be publicly available. Largely as the result of NSF's forward thinking program, many independent online resources for plant research have been developed in the past 12 years. While this abundance of genomic data has transformed plant science in many ways, it has also created some problems: the plethora of independent websites requires researcher awareness of the various projects and what data each offers. Finding and using these resources is not always straightforward. Most sites use a variety of different tools that are often unique to that resource, each requiring that the researcher learn how to interact with them. In addition, it is also often difficult to use the results from one resource in another, and it is not generally possible to search multiple resources at the same time. Instead, researchers find themselves repeating the same search (e.g., BLAST [1]) at multiple sites in the hopes of locating all information relevant to their research. In addition, when funding for a project ends, the data generated often are not moved to long-term repositories. Thus, project sites degrade over time and sometimes disappear entirely. When the previously accessible data disappear, generated resources are effectively lost. Aggravating these problems is the sheer volume of data available. These problems have been acknowledged by various groups, including the maize research community (reviewed in the 2007 Allerton Report at http://www.maizegdb.org/AllertonReport.doc) and are currently prevalent in many research communities [2–4].

Internet technologies evolved to accommodate the massive quantity of emerging genomics data and to deliver human-readable content of the ever-increasing amount of information. However, machine readability of this content lags dismally behind. Efforts have been underway for more than a decade to improve the machine readability of new types of data with the overarching goal of creating web resources that use standard ontologies that can be processed by machines. Most notable of these are technologies developed under the umbrella of the Semantic Web (http://www.w3.org/2001/sw/) such as the standard model for data interchange called RDF (Resource Description Framework; http://www.w3.org/RDF/) and a mechanism to process the data content called OWL (Web Ontology Language; http://www.w3.org/TR/owl-features/). Although improvements that make content more visual and accessible to humans have been widely adopted, new technologies and standards that enable machine-readable content have been adopted more slowly. Finding relationships, setting standards, and aggregating the complexities introduced by diverse data types is a challenge that has received a great deal of attention. Beavis [5] points out several issues that providers of biological data must address. Indeed, consortia of researchers focused on developing and implementing standards that have been formed including the Genomic Standards Consortium and the Genome Reference Consortium, and an open access journal called *Standards in Genomic Sciences *(http://standardsingenomics.org/) was founded in 2009. These efforts are actively ongoing especially in the life sciences and are gaining momentum, but at this time are not yet adequate for widespread implementation. The number and variety of rapidly evolving efforts for creating common standards is a challenge in its own right.

The overall objective of the POPcorn (PrOject Portal for corn; http://popcorn.maizegdb.org/) project is to develop unified public resources that facilitate access to the outcomes of maize genetics and genomics research projects and to ensure their sustainability by migrating them to MaizeGDB, the maize Model Organism Database (MOD) [6, 7]. POPcorn was designed from the outset to be a 2-year project with specific goals. Because of its short time frame, we did not aim to develop new technologies and solutions but rather to make use of the best existing technologies. This short time frame also forced us to create practical goals. Instead of locating and aggregating data from distributed resources, POPcorn allows access to distributed datasets primarily by sequence queries rather than by classifications or keywords. The advantage of this approach is that it does not require cross-compatibility of often idiosyncratic terminology. Rather, it focuses on a universal feature of genetic and genomic datasets: sequence. No matter how sophisticated or powerful, a resource is not useful unless it is adopted by researchers. For this reason, our primary goal has been to produce a resource that researchers will use to aid their discoveries.

### 1.2. Definitions

In order to describe the work accomplished as a part of the POPcorn project, a few definitions and discussions of the available technologies are in order:

**Provenance**

Where and by what means an item originated and the changes that may have occurred as the item moved from one place to another.

**Resource**

A database, data visualization, data search, data analysis, or any web application that serves or processes information.

**Sequenced-Indexed Data**

Data that are associated with sequence.

**Unlike Data**

Data in different formats and/or describing different things.

**Web Service**

A software system to support communication and data transfer among web resources, typically using a standard protocol such as the Representational State Transfer called REST [8] or the Simple Object Access Protocol, SOAP (http://www.w3.org/TR/soap12-part1).

## 2. Materials and Methods

### 2.1. Implementation

The POPcorn webpages were modeled after the online database PGROP (Plant Genome Research Outreach Portal; [9]), written in PHP and run on an Apache web server on a virtual machine created by VMware and running Red Hat Enterprise Linux Server release 5.6. The backend data processing scripts were written in Perl 5.8. The MaizeGDB Oracle 11 g database is accessed directly via SQL. Web services employed include wwwBLAST (Perl), NCBI's URLAPI, and a SOAP Web service for BLASTing was developed by PlantGDB (Java) [10]. Data were passed between scripts and services using XML (http://www.w3.org/TR/2008/REC-xml-20081126/) and JSON (http://json-schema.org/).

### 2.2. Development Approaches

In order to provide access to projects, project data, and web resources with a hand-curated, searchable database, content had to be loaded into the POPcorn curation database. These data are updated to the production site's database on the first Tuesday of each month. Currently, 242 project and resource descriptions are made available via POPcorn. Where possible, resources and data are associated with projects, and projects are related to one another where such a relationship makes sense. For example, the Maize Genome Sequencing pilot project (http://www.broadinstitute.org/annotation/plants/maize/) to evaluate strategies for producing a sequence of the Maize genome and to generate genome resources for the community is related to the funded B73 Maize Genome Sequencing project (http://genome.wustl.edu/genomes/view/zea_mays_mays_cv._b73) to sequence the gene space of *Zea mays *ssp*. mays* using the public B73 line. Researchers can submit their projects and correct the information residing at POPcorn via email or using links from the POPcorn website. Most projects were identified by POPcorn curators and developers by searching funding awards, attending conference talks, and viewing posters. Other projects and descriptions were provided by researchers directly.

One of POPcorn's objectives was to enable BLAST searches of multiple target datasets that are distributed across multiple websites from a single page. We used Web services to provide access to these datasets. In addition, distributing the BLAST requests via Web services permits multiple simultaneous BLAST jobs to execute on multiple servers. Most BLAST Web services were implemented with NCBI's wwwBLAST. One team (BioExtract; [11]) created a custom BLAST Web service for us. We also used NCBI's URLAPI Web service to run BLAST jobs on the NCBI servers against the most current GenBank data. We adapted CViT software (http://sourceforge.net/apps/mediawiki/cvit/index.php?title=Main_Page; [12]) to display sequence BLAST hits on the overall view of the B73 reference genome assembly.

Because POPcorn makes heavy use of Web services over which we have little control, an automated script checks all Web services daily and reports if any are not responding. Searches and BLAST targets that use Web services all check those services before appearing on pages in option lists. To the extent possible, errors that come from the Web service (e.g., a query too large for the BLAST service) are reported back to the researcher.

To create useful workflows, for example, “locate mutant seed stock containing variations in a gene of interest,” the multistep process was implemented in code to permit repetition of the same series of steps. The topic of workflows has received much interest of the past decade: systems like Taverna [13] and BioExtract [11] have been developed to enable users to create their own workflows for retrieving and analyzing data. The limited scope of the POPcorn project did not allow us to implement user-designed workflows. Instead we chose to “hard code” workflows for what we knew to be common tasks. Since POPcorn was charged to provide a sequence-based search resource for maize researchers; we call this collection of workflows the “Sequence-Indexed Data Search.” In designing tools that enabled researchers to carry out common tasks, we learned through discussions with researchers that they frequently begin a given task using keyword searches via GenBank's Entrez search service to find sequences. To incorporate this ability into POPcorn, we added a utility that accesses Entrez as a Web service.

### 2.3. Availability

The data, database, and code that make up POPcorn are in the public domain and are freely available upon request using the “Ask a Question” link at the top of any POPcorn page.

## 3. Results

### 3.1. Accomplishments

In developing POPcorn, we addressed four specific problems: (1) inability to locate all projects with data relevant to a particular research problem, (2) repetitive nature of performing the same sequence searches at multiple sites, (3) challenges associated with locating all types of data related to a particular sequence, and (4) issues associated with long-term data storage once individual projects have been completed.

#### 3.1.1. Search for Relevant Projects

In a rapidly evolving research area such as maize genomics and molecular biology, it can be particularly challenging to keep abreast of molecular tools and resources that can accelerate one's research program. Indeed, many have experienced the frustration of choosing a research path or approach, only to have it trumped or rendered less than cutting edge by the newest technological improvement. Since its inception in 1998, NSF PGRP has supported a rich variety of maize genomics projects, each developing useful tools and having its own project website. While this has moved the field forward by leaps and bounds, it is sometimes difficult to keep abreast of new and potentially useful advances. Most researchers would like to keep current with all the various research projects going on in their field in as efficient a way as possible.

To address this need for the maize and plant biology research communities, the Project Search feature (http://popcorn.maizegdb.org/search/project_search/project_search.php) at POPcorn was created. The Project Search accesses a hand-curated database of maize research projects and resources that is updated monthly and provides maize and plant biology researchers a one-stop shopping resource with reasonable assurance that it contains all publicly available maize resources and tools. To date, POPcorn enables access to 109 projects and 133 resources. Projects and resources are searched as separate entities and can be queried by keyword, investigator, institution, country, and category. Projects also can be accessed by browsing from five precompiled categories: sequencing, mapping, mutation, bioinformatics, and breeding. Given the complementary approaches that exist for searching POPcorn, virtually any approach a researcher might take towards locating a project or resource is likely to yield meaningful information.

#### 3.1.2. Simultaneous Sequence Search at Multiple Websites

One of the initial impacts of the PGRP was the rapid increase in the number of available maize DNA sequences from a wide variety of projects, each with its own particular biological focus. Initially many of the DNA sequences were expressed sequence tags (ESTs) from a wide range of tissues, genetic backgrounds, and treatments, each chosen to meet the specific needs of a particular project. Later, projects focused on genomic sequencing often with the goal of capturing the maize gene space because the Maize Genome Sequencing project [14] was not yet underway. Each of these project types generally made their sequencing results available through a project website prior to publication with eventual submission to GenBank. In many cases, projects generated and/or assembled sequence-associated information that could not be adequately represented and queried at GenBank. Maize and plant biology researchers often found themselves migrating from website to website, mining each for what it could contribute to their research. While the approach was workable for a very small number of projects generating small sequence sets, it quickly became burdensome for researchers to search many projects that housed large sequence datasets. It was especially difficult to compare results from the different sites side-by-side because each website used different parameters by default and displayed customized displays for result sets.

To address this problem and to allow a more rational and focused approach to searching maize sequence resources, POPcorn BLAST (http://popcorn.maizegdb.org/search/sequence_search/home.php?a=BLAST_UI) was developed. This utility permits BLAST searches of sequence resources at multiple sites using a single query. Datasets are searched directly at the host site with host tools or mirrored at MaizeGDB and kept current with update scripts that run at regular intervals. Researchers can upload individual sequences or batch files and search all or any combination of multiple databases (as of this writing, 44 BLAST targets are available), each with its own unique focus on a class or type of maize DNA sequence. Results for each of the databases can be viewed individually. Results are returned either by email or via web interface with a choice of multiple download formats.

#### 3.1.3. Finding Data Associated with Sequence

We developed a set of utilities that carry out multistep searches for sequence-indexed data, that is, data that can be found via sequence (expression patterns, similar sequences, functional annotations, associated locus, phenotype, traits, publications, etc.). For a detailed example of how these tools work, see “4. Example Usage Case: Sequence-indexed Data Search.”

#### 3.1.4. Migrating Data from Completed Projects to MaizeGDB

One unanticipated issue arising from the rapid proliferation of maize genomic tools and resources was a need to maintain generated data after completion of any given project. Although GenBank is tasked with maintaining the sequence information generated by a project, much of what these projects produce is beyond the scope of GenBank's mission. The vast majority of maize genomics projects are extramurally funded for two to five years. While funded, most of these projects do a good job of making their resources (either informational or physical) available to the maize research community. But what happens to these resources, some developed at considerable expense, when project funds for supporting them have been exhausted? Many projects manage to maintain and distribute resources for a period of time, but ultimately their ability to do so declines and valuable resources can be lost. To address the issue of potential information loss, the POPcorn project developed the ZeAlign tool (http://zealign.maizegdb.org/) to prepare sequence-indexed data for inclusion in MaizeGDB, the final repository for maize data and for the tools and processes developed as a part of the POPcorn project. ZeAlign enables researchers to align their sequences to the latest maize reference genome assembly using BLAST, then submit their alignments to MaizeGDB for public display via the MaizeGDB Genome Browser [15]. In addition to new tools, project records maintained by POPcorn include project expiration dates so that the MaizeGDB team knows when to begin contacting PIs to obtain data that should be brought into MaizeGDB.

### 3.2. Data, Methodologies, and Tools

Over the course of developing POPcorn, various aspects of data aggregation, query technologies, and standard terminologies were considered. In many cases, selections of particular methodologies and tools were made based upon research needs combined with practicality. Those decisions and selections are discussed in the subsequent sections.

#### 3.2.1. Data

One common approach to aggregating unlike data is to combine it all within a single relational *data warehouse*. This provides good control over the quality and structure of the data, but costs a great deal in terms of curator time. Another approach is to link databases into a network of *federated databases*. This distributes the responsibility for maintaining data, but limits access and control over the quality and structure of the data.

POPcorn does not itself catalogue research data; instead it contains information describing data (metadata) and how to access available resources. We were able to make use of MaizeGDB and NCBI [16] warehoused data to search and retrieve information from many projects and to access additional databases: GRASSIUS [17], Gramene [18], MaizeSequence.org (http://www.maizesequence.org/), DFCI [19], Plant Genomics MAGIs [20], PLEXdb [21], PlantGDB [10], the Photosynthesis Mutant Library (PML) [22], and Phytozome (http://www.phytozome.net/search.php). Personnel working at these databases allowed access to their data and, in some instances, installed or permitted us to install Web services on their servers (GRASSIUS, DFCI, PLEXdb) or developed tools (PlantGDB) to enable our access.

#### 3.2.2. Connecting Offsite Resources to the POPcorn Project

Similar to data, tools may be maintained locally or distributed and accessed by various technologies including various Web services. Where possible we have used existing technological solutions to the problems of distributed data and resources, such as Web services and two machine-readable protocols for encoding data, XML and JSON. We chose not to use more sophisticated technologies like SSWAP (Simple Semantic Web Architecture and Protocol) [23, 24] or SPARQL (SPARQL Protocol and RDF Query Language; http://www.w3.org/TR/rdf-sparql-query/) because, although they show promise, there is less likelihood of their long-term success and their current implementations were too limited for our needs. Data available via these technologies tend to be to proof-of-concept implementations or limited in scope. We had hoped to make considerable use of Web services to search and retrieve data from collaborator databases, but we found this approach challenging to implement and not robust. POPcorn has little to no control over Web services provided by other websites, especially in managing, recovering from, and reporting errors back to the researcher. Most of the Web services we found that best suited our needs were those provided by NCBI: eUtils (which we used for searching NCBI Entrez databases; http://www.ncbi.nlm.nih.gov/books/NBK25501/), URLAPI (which we used for executing BLAST searches at NCBI; http://www.ncbi.nlm.nih.gov/staff/tao/URLAPI/new/index.html), and wwwBLAST (which is freely available for download and can be installed as a BLAST Web service on any database server; http://www.ncbi.nlm.nih.gov/staff/tao/URLAPI/wwwblast/). We requested that wwwBLAST be implemented at some of our collaborator websites, including GRASSIUS, DFCI, and the Plant Genomics MAGI site, and we installed wwwBLAST at PLEXdb. We also made use of a BLAST Web service developed by PlantGDB's BioExtract team to execute BLAST searches against datasets at PlantGDB.

#### 3.2.3. Identifiers

Attaching unique, unambiguous identifiers to sequences has long been challenging. For example, all sequences at NCBI have an accession number (e.g., AF448416 is the accession number for the genomic sequencing containing the maize *bronze1 *locus), a version extension for the accession (e.g., AF448416.1 is the first version of the sequence record and AF448416.2 would represent the second version), and a GI number unique to each version of an accession (e.g., AF448416.1 is assigned GI 18092333, and subsequent versions of the accession would have an entirely unique and unrelated new GI designation assigned). This topic is discussed more fully at NCBI (http://www.ncbi.nlm.nih.gov/Sitemap/sequenceIDs.html). We decided to use mainly GenBank accessions for identifiers because the GenBank accession number without a version appended identifies the most up-to-date version of a given sequence. Where no GenBank identifier exists, we use project-specific identifiers. For example, for the most up-to-date maize gene model sequences we use the Gramene gene model names (*bz1*'s gene model identifier is GRMZM2G165390), both because the availability of those sequences from GenBank lags behind their public release and because researchers use those identifiers frequently in their own research.

#### 3.2.4. Locating Resources

POPcorn's projects and resources were located and curated into the database by hand. It was not until the site served quite a few project descriptions and resources (~100 total) that outside groups began to contact us directly to request specific changes to their own projects' descriptions and to request inclusion of new projects and resources.

#### 3.2.5. Divergent Data Types

POPcorn did not attempt to tackle the problem of integrating divergent data types (also called “unlike data”) except to show all related data located during sequence-based, multistep data searches (called sequence-indexed data searches; described below). To establish relationships between classes of data, POPcorn relied on human curation rather than formalized data descriptions. Indeed, formalized data descriptions did not exist for the majority of the data we accessed.

#### 3.2.6. Verifying Links

One problem with the ubiquitous links in webpages is not knowing what is on the other end of a link or if anything is even there at all. POPcorn addressed the former with hand curation and the latter with an automated script that runs daily to verify that all webpages and Web services listed in the database are responding as expected. Missing pages and services are reported to the POPcorn team for remedy and data searches that rely on missing Web services are taken offline until the problem is corrected. In addition, pages that present more than one search type to the researcher and involve one or more Web services are verified before the pages loads in the browser and options are presented.

#### 3.2.7. Versioning and Provenance

A significant problem associated with gathering data from distributed sources is the risk of the “provenance data” being lost along with accompanying credits and citations. Provenance data are a record of the workflow activities invoked, services and databases accessed, datasets used, and other specifics of the computational analysis detailing how, where, when, why, and by whom the results of an experiment were generated [25]. POPcorn strives to give credit to the data providers by providing extensive information in the project pages and, where possible, also gives data version information.

## 4. Example Usage Case: Sequence-Indexed Data Search

Researchers often want to identify mutant alleles to characterize the function of a gene or set of genes of interest. For example, once a gene or sequence of interest is identified, the characterization of additional alleles can confirm gene function: multiple alleles demonstrating a shared phenotype are excellent confirmation that a particular gene is responsible for a given phenotype. Indeed, characterization of multiple alleles of a gene is often required by journals for publication. For maize, there are several publicly available collections of mutants with transposable element insertions in known locations. POPcorn enables researchers to search various databases simultaneously for sequences of interest that harbor a mutation within or near the query sequence. Outputs contain not only the sequence itself but also identifiers for and access to relevant seed stocks.

In our example, a researcher we will call “Jane Smith” is working to increase the density at which plants can grow and has identified a sequence that confers an “upright leaves” phenotype in some maize lines that could be useful (reviewed in [26]). She would like to determine whether anything about this sequence has been published and if there are publicly available stocks that contain mutations in this sequence and, if so, obtain the seed stocks for her own investigations. To accomplish these goals, Jane goes to the POPcorn homepage: http://popcorn.maizegdb.org/main/index.php, then selects the “Search maize resources associated with a sequence” button under the “Sequence → Biology” header.

**Figure 1:**
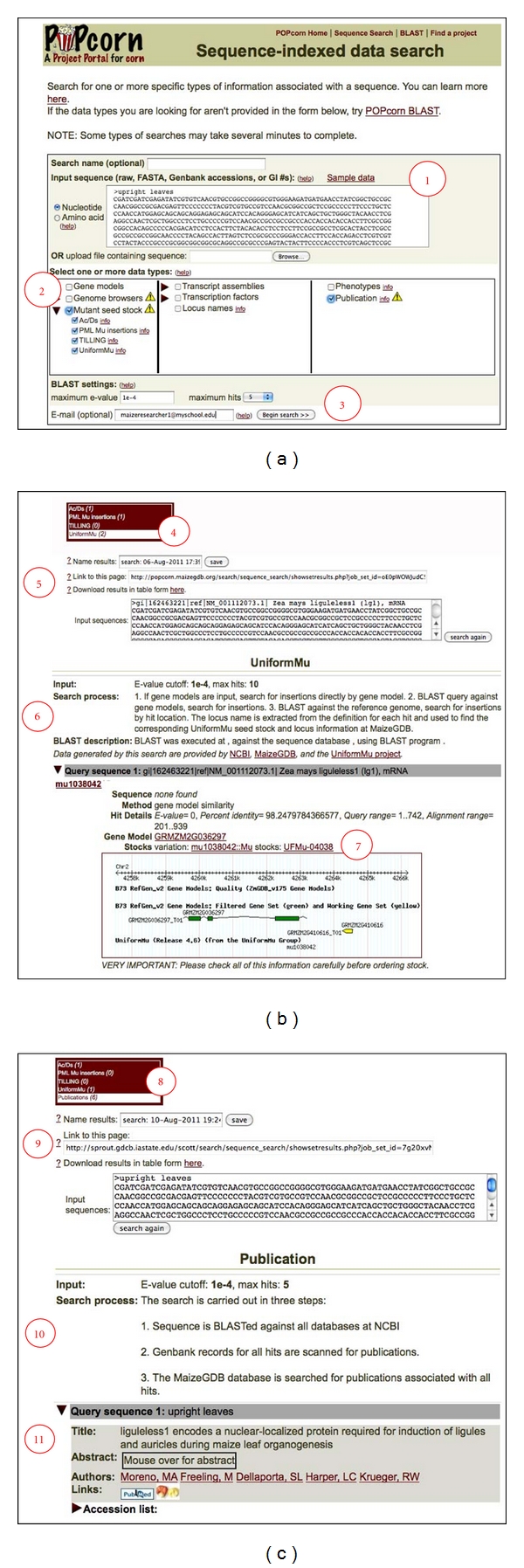
Sequence-indexed data search: sequence to stocks and publications. To locate stocks with mutations in or near the sequence of interest as well as relevant publications simultaneously, the process consists of steps demarcated by red circles. (a): (1) Paste a sequence (FASTA-formatted or raw) or sequence identifier (GenBank accession number or GI) into the “Input sequence” field and indicate whether the sequence is made up of nucleotides or amino acids. (2) Next, select one or more datasets to search. Here, mutant seed stocks and publications are selected. (3) Type an email address into the text box (optional) then click the “Begin search” button. (b): (4) The number of results from each search type are shown. Once a results set has been chosen (here “UniformMu”), (5) BLAST parameters used to produce the results are displayed. (6) The algorithm for conducting the selected search is shown, and (7) identified gene models and stocks are listed along with a snapshot of the MaizeGDB Genome Browser in the region of the mutation to show genomic context. (c): (8) The number of results from each search type is shown. Once a results set has been chosen (here “publications”), (9) BLAST parameters used to produce the results are displayed. (10) The algorithm for conducting the selected search is shown, and (11) identified citations are shown with links to both PubMed and MaizeGDB.

On the next page (Figure 1), she pastes the DNA sequence of her gene of interest and selects checkboxes to search both “Mutant seed stocks” and “Publications.” These selections enable simultaneous searches of relevant publications (indexed at both NCBI's PubMed and MaizeGDB) as well as sequence-indexed insertions of *Mutator *(*Mu*) elements [27, 28], *Activator/Dissociation* (*Ac*/*Ds*) elements [29–32], and TILLING point mutations [33] for which seed stocks are available. When she clicks the “Begin search” button, POPcorn carries out the following steps.

*First*, the input may be FASTA or raw sequence, GenBank IDs, or Gramene maize gene model IDs. If needed, the sequences associated with identifiers are retrieved and all is converted to FASTA.

*Next*, the following five searches are carried out simultaneously.

1. UniformMu
  (1.1) If input is one or more gene model names, look up UniformMu insertions directly in the MaizeGDB database and go to step 1.7.
  (1.2) If input is a GenBank identifier, request the FASTA-formatted sequence from GenBank via Web service and go to step 1.3.
  (1.3) BLAST query sequence against the current working gene set to get the gene model name(s) of the sequence [14]. If there is a matching gene model, go to step 1.4. If no gene model is returned, go to step 1.5.
  (1.4) Do a direct lookup in the MaizeGDB database using the gene model names. If successful, go to step 1.7.
  (1.5) BLAST query sequence against the current reference genome assembly.
  (1.6) Get hit coordinates, add 200 upstream and downstream bases, then do a direct lookup in the MaizeGDB database.
  (1.7) Get locus record at MaizeGDB.
  (1.8) Get variation records for that locus at MaizeGDB.
  (1.9) Get UniformMu stock records for each variation at MaizeGDB.
2. *Ac/Ds*
  (2.1) BLAST at GenBank against flanking sequence for *Ac/Ds*.
  (2.2) Search for *Ac/Ds* record at MaizeGDB using hit accession.
  (2.3) Generate links to PlantGDB for viewing insertion information and ordering stocks.
3. PML *Mu* insertions
  (3.1) BLAST at MaizeGDB against current filtered gene models.
  (3.2) Use gene model IDs to link to the PML *Mu* insertions website to get insertion and seed stock information.
4. TILLING
  (4.1) BLAST at GenBank against the Maize TILLING project target sequences.
  (4.2) Search for associated locus record at MaizeGDB.
  (4.3) Search for stock record associated with the locus record.
5. Publications
  (5.1) Execute simultaneous BLAST searches against GenBank's protein, nucleotide, GSS, and EST databases.
  (5.2) For each hit, search MaizeGDB data for matching loci.
  (5.3) For each locus found, search for associated publications.
  (5.4) For each hit, check the GenBank record for publication information.

*Finally*, when all searches are completed, display results on one page with a tab control to enable viewing each results set.

For the example given, results appear directly within the web browser or, if Jane wishes to do other things while waiting for the searches to complete, using an output link that is sent to her via email once the results set has been generated (Figure 1(b)). For the search of maize stocks, there are several possible results. In this case, there are *Mu* and *Ac/Ds* insertions in the input sequence. Jane can use the provided links to carefully check the results to see if they are useful. For example, an insertion may be located in an intron and she may only be interested in insertions into exons. Another possible result is an insertion into a sequence that does not contain an established gene model. Lastly, it is possible that no hits will be returned for the query sequence: whereas there are ~6,500 *Mu* insertions and 2351 *Ac/Ds* insertions publicly available, there are currently over 39,000 well-supported gene models (i.e., the “Filtered Gene Set”) in maize [14].

Simultaneously, the results (Figure 1(c)) indicate that Jane has located 8 citations to her best hit: the sequence of the well-characterized *liguleless1* gene.

By using this sequenced-indexed data search, Jane has been spared several tedious steps and can quickly determine if there are available resources for characterizing the biological function of her sequence of interest. Equally important, Jane's chances of finding a mutation in her gene of interest are increased by using POPcorn because otherwise she would have had to locate all relevant project websites, which is not a trivial task. In addition, were she to perform the same search of all projects she could locate by hand, each would have its own biases and eccentricities based upon differing default parameters, which would make direct comparisons of results from diverse websites difficult.

## 5. Conclusions and Future Directions

The technologies used to approach research problems in molecular genetics and plant breeding evolve continually, with sequence becoming the true “coin of the realm.” Various online resources emerge over time, with little more than sequence in common. In order to best support the paradigm shift from the use of molecular markers to genotype by sequencing, online resources must be reworked to accommodate a more sequence-centric perspective.

In its current implementation, POPcorn serves as a centralized web-accessible resource for gaining access to outcomes of maize research projects with an emphasis on the use of DNA and protein sequence for query inputs. The underlying premise is that, by creating a single point of access, researchers save valuable time and incorporate resources previously unknown to them into their analyses. By design, working in tandem with MaizeGDB allowed for the development of a pipeline whereby ongoing projects' data are accessible via POPcorn during the projects' funded period and relevant data are simultaneously prepared for inclusion in MaizeGDB at the end of a project's funded period. In addition, by bringing collaborators' project data into MaizeGDB at the end of their funding periods, these valuable data are preserved on the long term.

Discussions with maize researchers always indicate that clear, easy-to-use graphical user interfaces (GUIs) are critical for success. Investigators found the sequence-indexed data searches to be very helpful, but many required assistance to perform the searches. Part of this difficulty was conceptual (very few search utilities permit searching multiple data types starting from sequence) and part was due to the GUI. This problem is being addressed in part by integrating the developed sequence-indexed data search into relevant pages at MaizeGDB as well as by modifying the GUI on the main POPcorn search page based on guidance from researchers using the tool.

We set out to make use of the most effective solutions available. All features described here are available via the POPcorn stand-alone website: http://popcorn.maizegdb.org/. This site will be maintained by MaizeGDB personnel, and features developed for POPcorn are currently being incorporated into the MaizeGDB website directly. For example, the search for UniformMu seed stock described above has been integrated into the page describing the UniformMu project. POPcorn BLAST will be merged with the MaizeGDB BLAST resource, and the coded workflows are being integrated as a unit. The POPcorn resource currently is accessed by 114 unique users per day (averaged over a 6 month period).

Going forward, new project and resource records along with new BLAST target databases and new sequence-indexed data searches will be added as the landscape changes. We will continue to monitor funding awards and attend conference talks and poster sessions to learn about projects that should be indexed at POPcorn. The addition of new data and projects to the BLAST databases and the sequence-indexed data search requires that collaborators open their data to POPcorn, typically through a Web service such as wwwBLAST, and work with the MaizeGDB team to ensure that the logical steps required for deploying a sequence-indexed data search of new data types are correct. Integration of developed technologies into the MaizeGDB resource likely will increase usage significantly. We hope that the success of our end product will be judged by its usability and adoption by the research community it seeks to serve.
